# Water-Extracted *Ganoderma lucidum* Induces Apoptosis and S-Phase Arrest via Cyclin-CDK2 Pathway in Glioblastoma Cells

**DOI:** 10.3390/molecules25163585

**Published:** 2020-08-06

**Authors:** An-Yi Cheng, Yi-Chung Chien, Han-Chung Lee, Yi-Hsien Hsieh, Yung-Luen Yu

**Affiliations:** 1 The Ph.D. Program of Biotechnology and Biomedical Industry, China Medical University, Taichung 40402, Taiwan; s2012120518@gmail.com; 2 Graduate Institute of Biomedical Sciences, China Medical University, Taichung 40402, Taiwan; hardway19800710@gmail.com; 3 Center for Molecular Medicine, China Medical University Hospital, Taichung 40402, Taiwan; 4 Drug Development Center, China Medical University, Taichung 40402, Taiwan; 5 School of Medicine, College of Medicine, China Medical University, Taichung 40402, Taiwan; D5870@mail.cmuh.org.tw; 6 Department of Neurosurgery, China Medical University Hospital, Taichung 40402, Taiwan; 7 Department of Biochemistry, School of Medicine, Chung Shan Medical University, Taichung 40201, Taiwan; 8 Clinical Laboratory, Chung Shan Medical University Hospital, Taichung 40201, Taiwan; 9 Department of Biotechnology, Asia University, Taichung 41354, Taiwan

**Keywords:** glioblastoma, *Ganoderma lucidum*, apoptosis, S phase arrest, CDK2

## Abstract

Glioblastoma is one of the most common and most aggressive brain cancers. The current treatment is mainly surgery, chemotherapy, and radiation therapy, but the results are not satisfactory. *Ganoderma lucidum* (*G. lucidum*), also called “Lingzhi”, is a medicinal mushroom that has been used as a therapeutic agent for the treatment of numerous diseases, including cancer. However, whether it is effective for treating cancer is still unclear. In the present study, the anti-tumor effect of a water extract of *G. lucidum* was investigated using brain tumor cells. We used an analysis of cell viability, flow cytometry, the IncuCyte live-cell analysis system, and Western blotting to study its effects. The water extract from *G. lucidum* inhibited cell proliferation in a dose- and time-dependent manner, and it induced mitochondria-mediated apoptosis and cell cycle arrest at S phase via the cyclin-CDK2 pathway in human brain tumor cells. In addition, the *G. lucidum* extract significantly inhibited cell migration and mesenchymal marker expression based on the IncuCyte live-cell assay and qRT-PCR analysis. In summary, these anti-tumor effects in brain tumor cells suggest that *G. lucidum* may be useful for treating brain tumors.

## 1. Introduction

Glioblastoma multiforme (GBM) is the most aggressive glioblastoma and has an annual incidence rate of 5.26 per 100,000 people per year; of the 17,000 individuals who are diagnosed each year, most have a poor prognosis and greatly reduced quality of life [1]. According to the World Health Organization, glioblastoma is the most common primary brain neoplasm. From 60% to 75% of astrocytic tumors are classified as glioblastomas [2]. Grade IV glioblastoma is the most malignant glioma. The standard treatment for diagnosed glioblastoma is to perform surgery when applicable followed by radiation therapy [3]. Radiotherapy seems to be only related to prolonged progression-free survival, as it can better control seizures, but there is no substantial difference in overall survival. In addition, patients with radiation therapy have a high risk of experiencing some complications, such as post-radiation leukoencephalopathy, which is characterized by dementia, gait disturbance, incontinence, and insufficient attention and executive function. Among patients with recurrent GBM, the progression-free survival at 6 months after being treated with temozolomide (TMZ) is only 21% [4,5]. The discovery of novel and effective agents for the treatment of GBM is, consequently, a pressing need. Natural products with their highly complex bioactivities and functions play an important role in drug discovery for treating diseases [6].

*Ganoderma lucidum* (*G. lucidum*) is one of the well-known traditional Chinese medicines among Eastern countries and has been used in China, Japan, Korea, and other Asian countries for more than 2000 years [7,8,9]. *G. lucidum* has been found to be an effective supplementary medicine for the prevention and treatment of various diseases in the past few decades. In addition, research in the last decade has shown that *G. lucidum* has a large number of pharmacological functions, such as enhancing immunity, antioxidant, anti-inflammatory, and anti-cancer effects. [10,11,12,13,14,15,16]. Moreover, it was reported that *G. lucidum* polysaccharides, which are one of the main effective components, can suppress tumor progression, such as melanoma, lung cancer, breast cancer, endometrial cancer, bladder cancer, and colorectal cancer [7,8]. Water extract of *G. lucidum* generally consists of polysaccharides, which inhibit colorectal cancer by promoting apoptosis or cell cycle arrest and reduce the gene expression which is responsible for cell proliferation [16]. In clinical trials, *G. lucidum* extract can also increase the survival rate of cancer patients [10].

Therefore, in this current study, we evaluated the anti-cancer effect of the water-soluble components of *G. lucidum* on glioblastoma and further investigated the possible mechanisms. The results suggested that water extract of *G. lucidum* induced apoptosis and inhibited the effect of migration in glioblastoma.

## 2. Materials and Methods

### 2.1. Preparation of G. lucidum Extract

The *G. lucidum* used in this study was provided by Taiwan Sungertain Ganoderma Farm (Taipei, Taiwan), which grinds the *G. lucidum* fruiting body into a powder. First, to avoid the osmotic pressure of water, 1 g of *G. lucidum* powder was dissolved in 10 mL RPMI-1640 medium (Hyclone) without serum and stirred (250 rpm) at 37 °C for 30 min in a water bath. The solution was centrifuged at 1000× *g* for 5 min. The supernatant was then collected and filtered through a 0.22 μm filter to remove bacterium. Finally, we had a 200 mg/mL stock solution at 100%. For the in vitro experiments, the stock solution *G. lucidum* was diluted in RPMI-1640 with 10% fetal bovine serum (FBS).

### 2.2. Cell Culture

The U87 was obtained from American Type Culture Collection (ATCC, Manassas, VA, USA). The GBM8901 was purchased from Bioresource Collection Research Center (Hsinchu, Taiwan). The GBM8901 and U87 were cultured in RPMI-1640 medium supplemented with 10% fetal bovine serum and 1% penicillin and streptomycin in a humidified incubator at 37 °C with 5% CO_2_.

### 2.3. Cell Survival

To determine cell survival, the GBM8901 and U87 cells were seeded in 96-well plates at a concentration of 5 × 10^3^ cells/well. On the next day, the medium was removed and replaced with fresh medium with or without *G. lucidum*. After 24, 48, and 72 h, cell survival was determined by using 3-(4, 5-dimethylthiazol-2-yl)-2,5-diphenyltetrazoliumbromide(MTT) (5 mg/mL, 20 μL per well) for 2–4 h at 37 °C. The absorbance was measured at 570 nm by an ELISA reader (BioTek Inc., Taipei, Taiwan). The percentage of viable cells relative to control cells without *G. lucidum* treatment was calculated.

### 2.4. Cell Cycle Analysis

For the cell cycle analysis experiments, the GBM8901 and U87 cells were seeded in 6 cm dishes (1 × 10^5^ cells/well) and incubated overnight before being treated with 10% *G. lucidum* extract for 24, 48, and 72 h. The cells were then washed with PBS and fixed with ice-cold 75% ethanol overnight at 4 °C. The fixed cells were centrifuged at 1500× *g* for 5 min; the alcohol wash was removed, and the cells were rinsed with PBS and then centrifuged at 1500× *g* for 10 min. After removal of the PBS wash, 50 μL of a 100 μg/mL stock of RNase was added. This ensured that only DNA, not RNA, was stained. Then, a propidium iodide (PI) solution (20 μg/mL in PBS) was added and incubated for 30 min at room temperature to stain. The cell cycle distribution of the cells was analyzed by a FACSverse Flow cytometer (BD Biosciences, San Jose, CA, USA) and the Mod Fit LT 4.1 software (Verity Software House, Inc., Topsham, ME, USA).

### 2.5. Annexin V/PI Staining

To analyze the GBM8901 and U87 cells, they were seeded in 6 cm dishes (3 × 10^5^ cells/well) and were incubated and treated with *G. lucidum* extract as described above for the cell cycle analysis. Cells were collected and stained with Annexin V-FITC/PI apoptosis kit (BioVision, Milpitas, CA, USA). First, 100μL of binding buffer was added, and then mixed with 5 μL of Annexin V and 5 μL of PI at room temperature in the dark for 15 min. Finally, 400 μL of binding buffer was added and then analyzed by flow cytometry as described above [17].

### 2.6. Isolation of Cytosolic Protein from Mitochondria

To separate cytosolic proteins from mitochondria, cells (10^6^) were lysed in 30 μL of ice-cold lysis buffer (80 mM KCl, 250 mM sucrose, 500 μg/mL digitonin and proteases inhibitors in PBS). Then, cell lysates were centrifuged for 5 min at 10,000 rcf. Proteins from the supernatant (cytosolic fraction) and pellet (mitochondria fraction) were collected for further experiments.

### 2.7. Western Blotting

For Western blotting analyses, the collected cells were rinsed in PBS and lysed in NETN lysis buffer (Bethyl Laboratories, Inc., Montgomery, TX, USA). Equal amounts of protein were separated on 10%–12% SDS-polyacrylamide gels, transferred to PVDF membranes, and blocked with 5% nonfat dry milk in TBST for 1 h. The membranes were then incubated with primary antibodies overnight at 4 °C. Antibodies against PARP, E-cadherin, N-cadherin, vimentin, cyclin A2, cyclin D1, CDK2, and β-actin were purchased from Cell Signaling Technology (Beverly, MA, USA) (all used at 1:1000 dilutions). Antibodies against cytochrome c were purchased from Santa Cruz Biotechnology (Dallas, TX, USA) (1:2000 dilutions). After being washed 3 times with TBST for 10 min, the membranes were incubated with appropriate secondary antibodies in TBST for 1 h. After several washes of TBST, the blots were developed by enhanced chemiluminescence (ECL) solution (Bio-Rad, Hercules, CA, USA); Western blots were quantified using ImageJ software (1.8.0_112, Windows).

### 2.8. Wound-Healing Assay

For the wound-healing assay, the GBM8901 and U87 cells were seeded in 96-well plates (6 × 10^3^ cells/well) and allowed to reach 80% confluency for 12–24 h at 37 °C. The monolayer was then scratched using 96 scrapers (Essen Bioscience, Ann Arbor, MI, USA) to produce a wound. Each plate was washed with PBS to remove detached cells, and fresh medium was then added that contained different concentrations of the *G. lucidum* extract. Images were captured prior to (0 h) and 24 h after addition of the extract by the IncuCyte live-cell analysis system (Essen Bioscience, Ann Arbor, MI, USA). The images were processed with the ImageJ software.

### 2.9. RNA Extraction and Real-Time PCR

To isolate RNA for real-time PCR, the GBM8901 and U87 cells were directly lysed by mixing with 1 mL of Trizol reagent and were homogenized. The GBM8901 and U87 cell lines were treated with *G. lucidum* for 24, 48, and 72 h, and then the cells were extracted using Trizol reagent (Invitrogen, Carlsbad, CA, USA). To each sample, 0.2 mL of chloroform was added. The samples were mixed and allowed to stand for 2–3 min and then were centrifuged at 12,000× *g* for 15 min at room temperature. The upper phase was transferred into a clean 1.5 mL tube and 0.6 mL of isopropanol was added, and the mixture was stored at −80 °C for 2 h. The mixture was then centrifuged at 12,000× *g* for 20 min. A pellet was expected to be visible at the base of each tube. The isopropanol was poured off and 1 mL of 75% ethanol in diethyl pyrocarbonate (DEPC) treated H_2_O was added. The mixture was mixed gently and then recentrifuged at 12,000× *g* for 5 min. The ethanol was then poured off and the pellets were allowed to air-dry. Then, approximately 15–25 μL of TE buffer was added to the RNA pellet. The total RNA was quantified by spectrophotometry. The sequences of the PCR primers for each gene are [18]: E-cadherin(forward:5′-CCACCAAAGTCACGCTGAAT-3′, reverse:5′-GGAGTTGGGAAATGTGAGC-3′), N-cadherin(forward:5′-GTGCCATTAGCCAAGGAATTCAGC-3′, reverse: 5′-GCGTTCCTGTTCCACTCATAGGAGG-3′), Vimentin (forward:5′-ATGAAGGTGCTGCAAAAC-3′, reverse:5′-GTGACTGCACCTGTCTCCGGTA-3′), GAPDH(forward:5′-ATGAGCCCCAGCCTTCTCCAT-3′, reverse:5′-GGTCGGAGTCAACGGATTTG-3′). A total of 1 µg of RNAs from each sample was synthesized into cDNA by using reverse transcriptase with 5× first strand buffer, 0.1 M DTT, 100 nM oligo(dT)20 primer, 10 nM dNTP, Rnase-OUT (40U/μL), M-MLV (Invitrogen, USA), and DEPC water. For real-time PCR, mRNA was first reverse transcribed into cDNA as described [19]. The PCR conditions were as follows: pre-incubation at 95 °C for 30 s; followed by 40 cycles of denaturation at 95 °C for 5 s; and annealing at 60 °C for 30 s. The real-time PCR was carried out with a SYBR Green (Bio-Rad, USA) in a Light Cycler 480 (Roche, Basel, Switzerland). Each sample was run in triplicate, and at least three independent experiments were analyzed. Relative quantification of mRNA levels was conducted using the comparative C_T_ method, with GADPH as the reference gene and using the formula 2^−ΔΔCt^.

### 2.10. Statistical Analysis

The data are shown as the mean ± standard deviation (SD). The differences were analyzed with a Student’s *t*-test relative to the control. A value of *p* < 0.05 was considered to be statistically significant.

## 3. Results

### 3.1. G. lucidum Extract Inhibits Cell Survival in Glioblastoma Cell Lines

**Figure 1:**
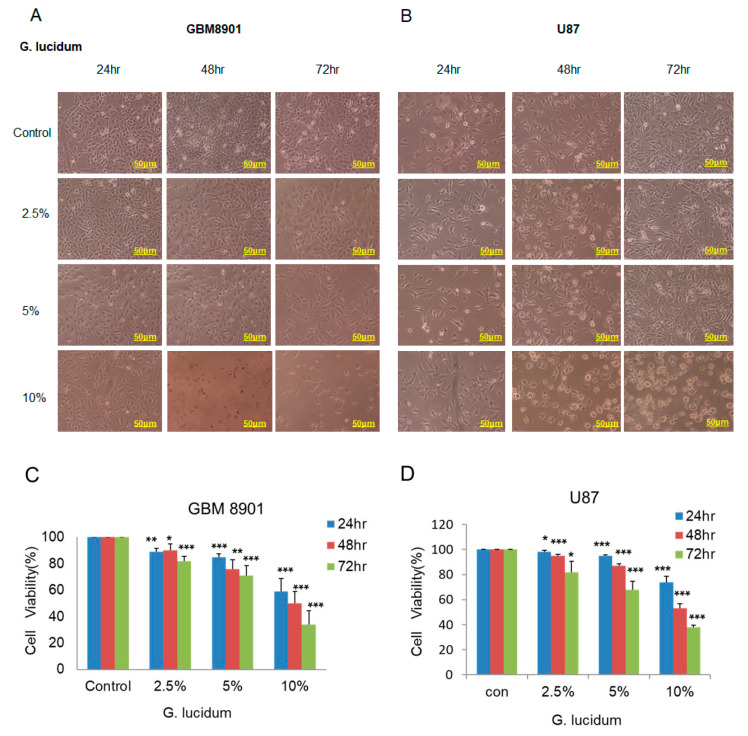
*G. lucidum* extract inhibits the growth of human glioblastoma cells. (**A**,**B**) The phenotype of (**A**) GBM8901 and (**B**) U87 cells treated with different concentrations of *G. lucidum* for 24, 48, and 72 h. (**C**,**D**) Cell survival of (**C**) GBM8901 and (**D**) U87 cells was examined using the MTT assay. * *p* < 0.05; ** *p* < 0.01; *** *p* < 0.001.

First, we used the MTT assay to investigate the effects of different concentrations of *G. lucidum* extract on GBM8901 and U87 cell survival after incubation for 24, 48, and 72 h. Cell survival was significantly decreased in a time- and dose-dependent manner in both glioblastoma cell lines (Figure 1A,B). After a 24 h incubation with 10% *G. lucidum* extract, cell survival decreased to 59% in the GBM8901 cells and 74% in the U87 cells. The inhibitory effect was further enhanced after 48 and 72 h treatments (Figure 1C,D), suggesting that longer treatment with *G. lucidum* might increase the inhibitory effects of mitochondria metabolic activity on the GBM8901 and U87 cells. Thus, the *G. lucidum* extract might have the ability to inhibit GBM cell lines proliferation.

### 3.2. G. lucidum Extract Induces Apoptosis in Glioblastoma Cells

**Figure 2:**
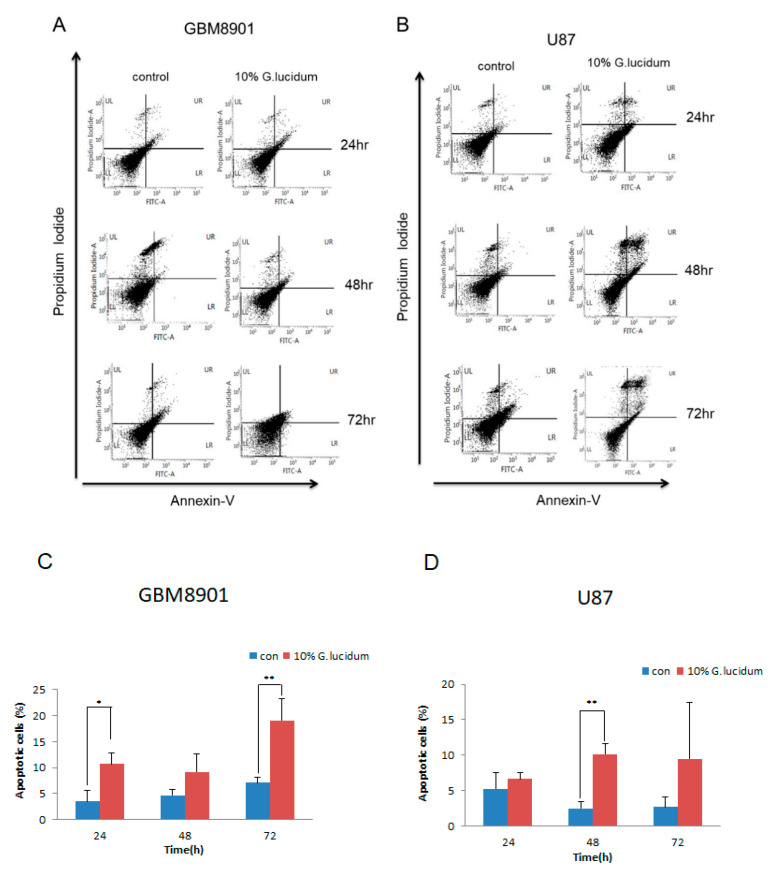

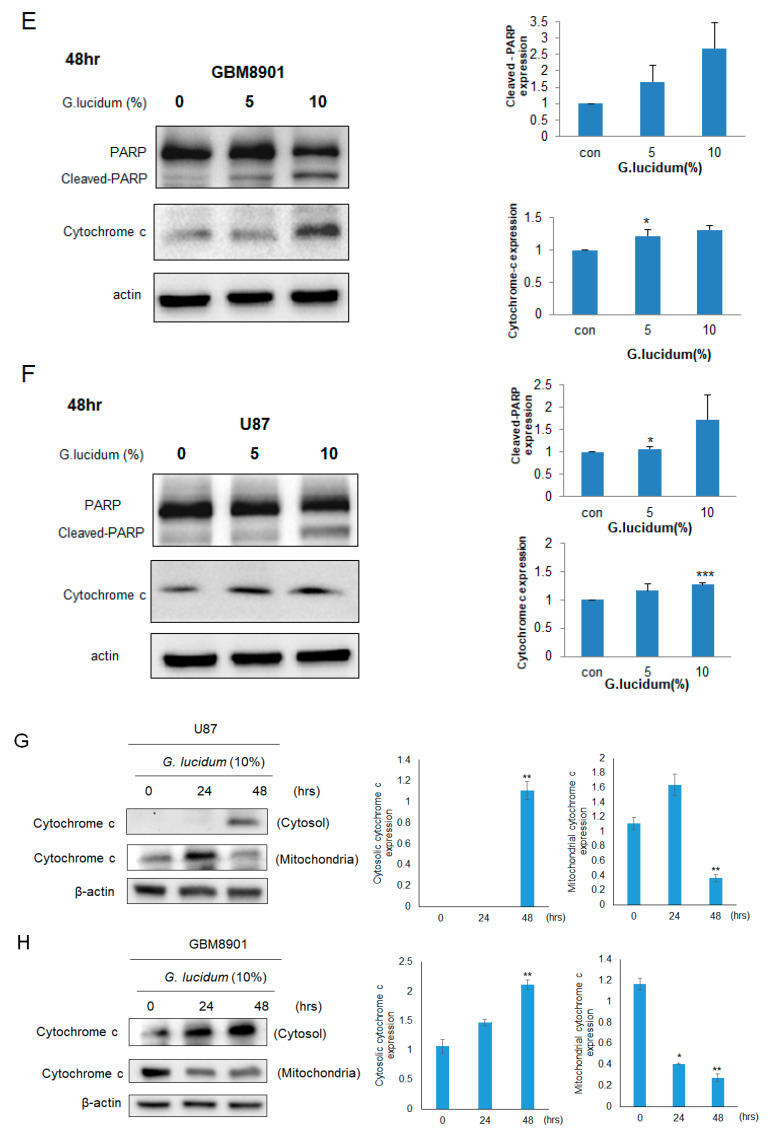
*G. lucidum* extract induces apoptosis in glioblastoma cells. (**A**,**B**) *G. lucidum* extract induces apoptosis of (**A**) GBM8901 and (**B**) U87 cells. (**C**,**D**) The straight bar chart shows the increase in apoptotic cells in the GMB8901 and U87 cells after treatment with *G. lucidum* in a time-dependent manner. (**E**,**F**) The expression of cell apoptosis-related proteins in (**E**) GBM8901 and (**F**) U87 cells as determined by Western blotting. (**G**,**H**) To detect cytochrome c release, cytosolic and mitochondrial fraction of (**G**) U87 and (**H**) GBM8901. * *p* < 0.05; ** *p* < 0.01; *** *p* < 0.001.

Using Annexin-V–FITC/PI double staining, we looked for apoptotic cells among the GBM8901 and U87 cells in cultures that were treated with 0 and 10% *G. lucidum* extract for 24, 48, and 72 h. The number of apoptotic cells in the treated GBM8901 and U87 cells increased in a time-dependent manner (Figure 2A–D). These results were confirmed by Western blotting in which cleaved PARP expression and cytochrome c release were increased in comparison to control levels (Figure 2E,F). Furthermore, we also analyzed the expression of cytochrome c in mitochondrial and cytoplasmic extracts. The release of cytochrome c in cytosol was enhanced within 0 to 48 h. In the mitochondrial fraction, we observed significantly reduced cytochrome c levels (Figure 2G,H). These results indicate that the *G. lucidum* extract induced apoptosis in the GBM8901 and U87 cells.

### 3.3. G. lucidum Extract Induces Glioblastoma Cells to Undergo Cell Cycle Arrest at S Phase

**Figure 3:**
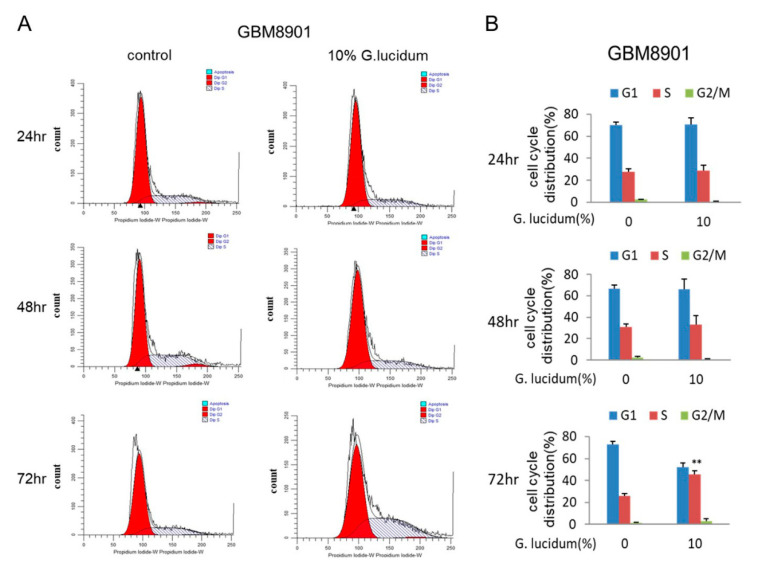

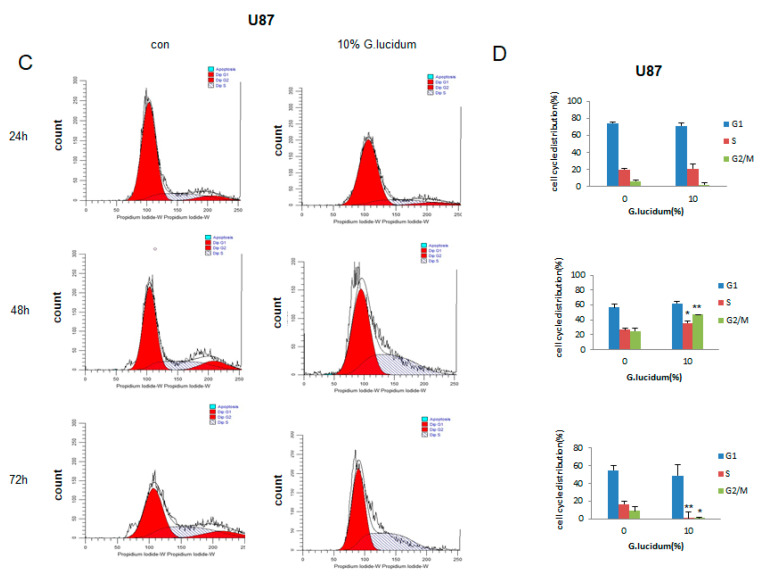
*G. lucidum* extract induces S phase cell cycle arrest in glioblastoma cells. (**A**,**C**) Cultures of (**A**) GBM 8901 and (**C**) U87 cells were treated with 10% *G. lucidum* extract for 24, 48, and 72 h, and the cell cycle distribution was assessed after PI staining by flow cytometry. (**B**,**D**) Cell cycle distribution of (**B**) GBM 8901 and (**D**) U87 cells. * *p* < 0.05; ** *p* < 0.01.

**Figure 4:**
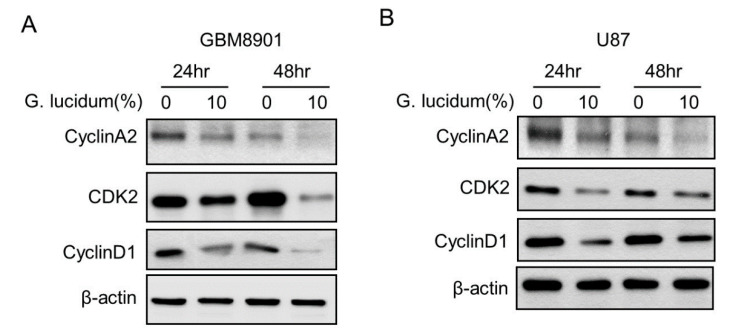

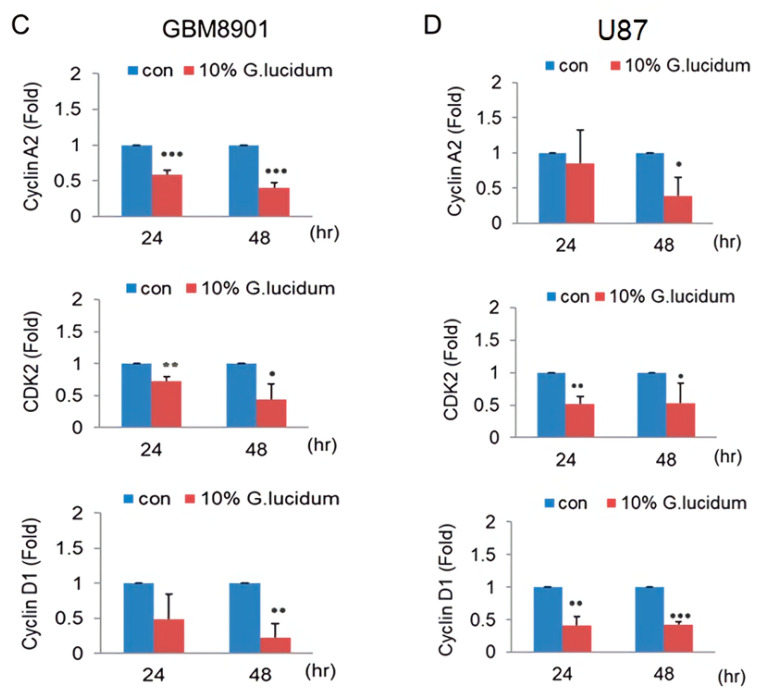
*G. lucidum* extract downregulates the expression of S phase-related proteins. (**A**,**B**) The expression of the indicated S phase-related proteins in (**A**) GBM8901 and (**B**) U87 cells was determined by Western blotting. (**C**,**D**) Quantification of protein expression from Western blots of (**C**) GBM8901 and (**D**) U87 cells as shown in A and B by ImageJ. * *p* < 0.05; ** *p* < 0.01; *** *p* < 0.001.

To evaluate whether *G. lucidum* could cause cell cycle arrest, we used flow cytometry analysis. The percentage of S phase cells in both the GBM8901 (72 h) and U87 (48 h) cultures was increased after treatment with 10% *G. lucidum* extract (Figure 3A–D). We confirmed these results by detecting the expression of cyclin A2, cyclin D1, and CDK2 proteins, all of which are essential for S phase [20]. After treatment with *G. lucidum* extract, the expression of cyclin A2, cyclin D1, and CDK2 proteins was decreased (Figure 4A–D). Together, these data indicate that *G. lucidum* extract induced glioblastoma cells to arrest at S phase by inhibiting the expression of cyclin A2, cyclin D1, and CDK2.

### 3.4. G. lucidum Extract Inhibits Cell Migration in Glioblastoma Cells

**Figure 5:**
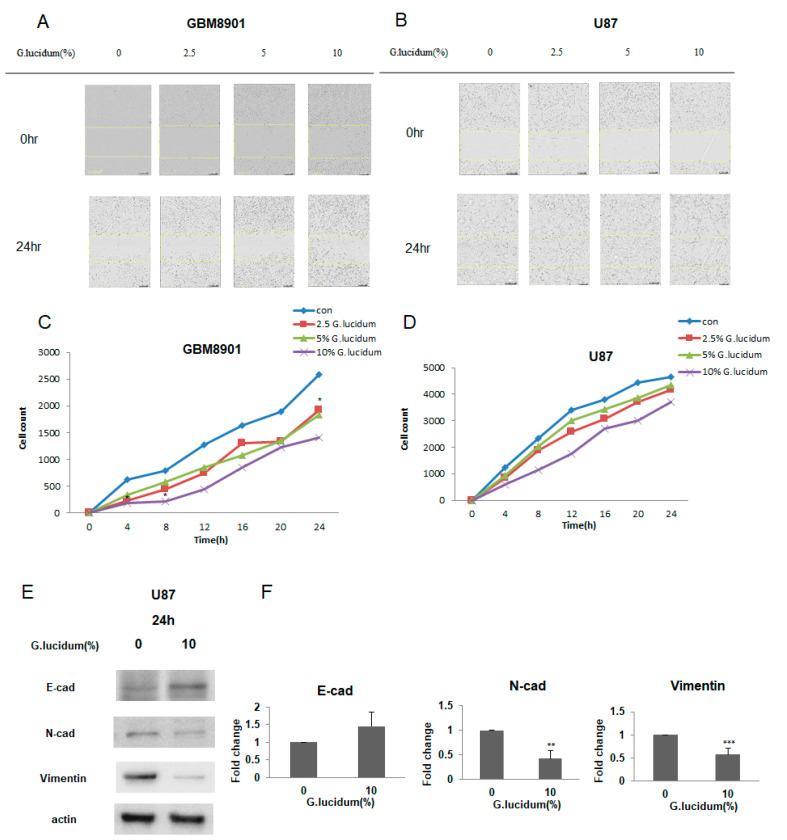
*G. lucidum* extract inhibits glioblastoma cell migration and inhibits the expression of epithelial–mesenchymal transition (EMT) markers in glioblastoma cells. (**A**,**B**) Cultures of (**A**) GBM 8901 and (**B**) U87 cells that had undergone wounding were treated with different doses of *G. lucidum* extract for 24 h. The extent of wound healing was then assessed with the IncuCyte system. (**C**,**D**) Quantification of (**C**) GBM8901 and (**D**) U87 cells that had migrated into the wound area. U87 glioblastoma cells were treated with or without 10% *G. lucidum* extract for 24 h. (**A**,**B**) The cells were then assessed for expression of E-cadherin, N-cadherin, and vimentin (**E**) at the protein level and at the mRNA level by qRT-PCR analysis (**F**) by Western blotting. * *p* < 0.05; ** *p* < 0.01; *** *p* < 0.001.

We next addressed whether *G. lucidum* extract can inhibit cell migration with a wound-healing assay using both the GBM8901 and U87 cells. After treatment with *G. lucidum* extract for 24 h, cell migration was inhibited, and this inhibition became more obvious with higher concentrations of extract (Figure 5A–D).

### 3.5. G. lucidum Extract Inhibits the Expression of Epithelial–Mesenchymal Transition (EMT) Markers on Glioblastoma Cells

As *G. lucidum* extract was able to inhibit cell migration, we next determined whether *G. lucidum* extract could also inhibit the expression of markers associated with EMT, a process involved in cancer cell metastasis [21]. We used Western blotting and q-PCR to investigate the expression of three EMT markers. E-cadherin is for epithelial marker. N-cadherin and vimentin are for mesenchymal marker. *G. lucidum* extract decreased the expression of N-cadherin and vimentin and increased the expression of E-cadherin (Figure 5E,F).

## 4. Discussion

Glioblastoma is the most aggressive primary cancer in brain tumors. Unfortunately, the treatment for GBM is very limited in clinical. The only chemotherapeutic treatment for GBM is TMZ, which is a DNA alkylating agent. However, the drug resistance to TMZ limits its clinical application. Therefore, a novel compound is desperately needed for improved treatment of GBM. In recent years, many studies of traditional Chinese medicines regarding their application in prevention and treatment of cancer have emerged [22]. The most widely used traditional Chinese medicine is *G. lucidum*. It has been considered as a medicine to improve immune function and facilitate longevity. Nowadays, we have a deeper understanding of *G. lucidum* and know that the major bioactive natural components of it are polysaccharides and triterpenes [23]. Some pre-clinical and clinical studies showed that *G. lucidum* has many medical effects, such as antioxidant, hypoglycemic, immune regulation, and anti-cancer [7,8]. According to reports, many biological compounds have been isolated from *G. lucidum*, which contain triterpenoids, polysaccharides, nucleosides, sterols, proteins, and alkaloids. Among these active ingredients, the more widely studied anti-cancer components are triterpenoids (active ingredients of *G. lucidum* through ethanol extract) and polysaccharides (active ingredients of *G. lucidum* through water extract). Because they are in the nutritional components of *G. lucidum*, polysaccharides and triterpenes are considered the most effective components. The results of the present study further reveal that polysaccharides may be the active compounds responsible for the anti-tumor effect of *G. lucidum*. However, how *G. lucidum,* a medicinal mushroom, affects human brain cancer cells is unclear.

In this study, we investigated the effects of *G. lucidum* extract on glioblastoma cells. Increasing concentrations of *G. lucidum* extract resulted in a significant decrease in cell survival in the GBM8901 and U87 cells as compared with the control (Figure 1C,D). Cytochrome c release is known to be a key event during mitochondria-dependent apoptosis. Therefore, we attempted to examine whether treatment with *G. lucidum* extract could enhance the expression of cytochrome c [24]. According to our data, apoptosis is increased in GBM cell lines by changes in the expression of proteins that promote this process. Moreover, the activation of caspase 3 cleaves regulatory proteins essential for cell survival and maintenance and cleaves poly (ADP-ribose) polymerase (PARP), which is involved in DNA repair and programmed cell death [25,26]. We used an Annexin V/PI staining assay with flow cytometry analysis to confirm that *G. lucidum* extract induced apoptosis in the GBM8901 and U87 glioblastoma cells as compared with untreated cells (Figure 2A–D). These results were confirmed by Western blotting, in which cleaved PARP expression and cytochrome c release were increased in comparison to control levels (Figure 2E–G).

Cell cycle disorder is one of the main contributors to carcinogenesis. Normal cells cycle through the phases G1, S, G2, and M, in that order, under the precise control of the cell cycle molecular network system [27,28]. Deregulation of the cell cycle results in an imbalance between cell proliferation and apoptosis, which may eventually lead to cancer. Our results suggest that *G. lucidum* extract can arrest the cell cycle at S phase as determined by flow cytometry in the GBM8901 and U87 cells. (Figure 3A–D). Both downregulation of cyclin A2 and CDK2 induced cell cycle arrest at S phase (Figure 4A–D) [29].

**Figure 6:**
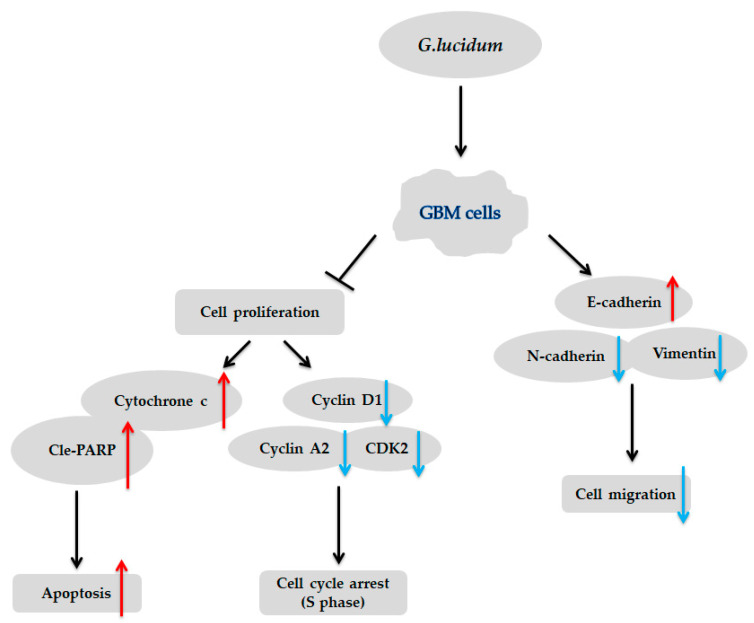
Proposed model for the mechanism of *G. lucidum* activity in human glioblastoma cells.

In addition, we found that *G. lucidum* extract inhibited cell migration, as determined by a wound-healing assay (Figure 5A–D), and this effect was associated with the upregulation of E-cadherin, which plays an important role in the process of cell adhesion and is downregulated in many tumors [30]. Consistent with our results, an earlier study reported that *G. lucidum* whole extract significantly inhibits breast cancer cell invasion by upregulating E-cadherin [31]. Taken together, these findings suggest that *G. lucidum* extract may inhibit the migration of glioblastoma cells, mainly by upregulating E-cadherin expression (Figure 5E,F). As *G. lucidum* extract can inhibit the proliferation and migration of glioblastoma cells by inducing apoptosis and cell cycle arrest, it may have therapeutic applications for the treatment of glioblastoma cells (Figure 6).
